# Recent advances in the role of high-salt diet in anti- and pro-cancer progression

**DOI:** 10.3389/fimmu.2025.1542157

**Published:** 2025-01-29

**Authors:** Shiwei Tang, Juan Xu, Ping Wan, Shumen Jin, Ying Zhang, Linting Xun, Jinli Wang, Mei Luo, Wenjie Chen, Zan Zuo, Hui Tang, Jialong Qi

**Affiliations:** ^1^ School of Medicine, Kunming University of Science and Technology, Kunming, Yunnan, China; ^2^ Yunnan Digestive Endoscopy Clinical Medical Center, Department of Gastroenterology, The First People’s Hospital of Yunnan Province, Affiliated by Kunming University of Science and Technology, Kunming, Yunnan, China; ^3^ Yunnan Province Clinical Research Center for Senile Diseases, First People’s Hospital of Yunnan Province, Kunming, Yunnan, China; ^4^ Yunnan Institute of Food and Drug Supervision and Control, Medical Products Administration of Yunnan Province, Kuming, Yunnan, China; ^5^ State Key Laboratory of Respiratory Disease, Guangzhou Medical University, Guangzhou, China; ^6^ Guangdong-Hongkong-Macao Joint Laboratory of Respiratory Infectious Disease, Guangzhou Medical University, Guangzhou, China; ^7^ Yunnan Provincial Key Laboratory of Clinical Virology, The First People’s Hospital of Yunnan Province, Kunming, Yunnan, China; ^8^ Yunnan Provincial Key Laboratory of Birth Defects and Genetic Diseases, First People’s Hospital of Yunnan Province, Kunming, Yunnan, China

**Keywords:** high-salt diet (HSD), immunotherapy, tumor microenvironment (TME), gut microbiota, sodium transporter channels

## Abstract

Dietary behaviors significantly influence tumor progression, with increasing focus on high-salt diets (HSD) in recent years. Traditionally, HSD has been regarded as a major risk factor for multiple health issues, including hypertension, cardiovascular disease, kidney disease, cancer, and osteoporosis. However, recent studies have uncovered a novel aspect of HSD, suggesting that HSD may inhibit tumor growth in specific pathological conditions by modulating the activity of immune cells that infiltrate tumors and enhancing the effectiveness of PD-1 immunotherapy. This review focused on the duel molecular mechanisms of HSD in cancer development, which are based on the tumor microenvironment, the gut microbiota, and the involvement of sodium transporter channels. The objective of this review is to explore whether HSD could be a potential future oncological therapeutic strategy under specific situation.

## 1 Introduction

According to the Global Cancer Observatory (GLOBOCAN) statistics for the year 2022, approximately 20 million new cancer cases were reported worldwide. It is estimated that approximately one in five individuals, regardless of gender, will develop cancer during their lifetime. Specifically, approximately one in every nine males and one in every twelve females will ultimately succumb to cancer (1). Furthermore, 90% of cancer deaths are attributed to metastasis (2–4). The development of cancer is the result of a complex interplay between risk factors and genetic or epigenetic changes, which provide cells with a selective advantage, allowing them to evade immune surveillance and undergo malignant transformation (5). Risk factors for cancer can be broadly classified into two categories: exogenous and endogenous. Exogenous factors include, but are not limited to smoking (6), an unhealthy diet (7), being overweight, lack of physical activity (8) and the microbial environment (9). Endogenous factors, on the other hand, include genetic susceptibility (10), individual DNA repair capacity (11). Poor dietary habits are considered one of the most dangerous exogenous factors that play a crucial role in the progression of cancer. Studies have shown that a high-fat diet can facilitate cancer progression through the activation of the mTORC1 signaling pathway mediated by the intestinal microbiota in myeloid progenitor cells (12).The French NutriNet-Santé prospective cohort study found that high-sugar diets are also strongly associated with cancer development,particularly in the context of breast cancer (13). The ketogenic diet activates AMP-activated protein kinase (AMPK), which in turn stimulates tumor suppressor genes such as p53 and LKB1. This process inhibits cell proliferation, mitigates inflammation, and restrains cell growth (14). The Mediterranean diet is abundant in monounsaturated fatty acids, notably oleic acid (OA). Research has demonstrated that oleic acid mitigates oxidative stress and inflammation (15). In conclusion, dietary patterns have a significant influence on the cancer development. Adopting healthy eating habits may be an effective way to prevent cancer, while unhealthy eating habits may increase the risk of cancer.

HSD has previously been identified as a potential risk factor for tumor formation. This is largely due to its ability to induce a chronic inflammatory microenvironment (16), which in turn stimulates continuous cell proliferation, DNA damage and cancer transformation (17) For instance, the released IL-17 activates the MAPK/ERK signaling pathway, promoting the proliferation, migration and invasion of human breast cancer cells (18). HSD has been demonstrated to enhance cancer cell proliferation and metastasis. High salt exposure activates salt-inducible kinase-3 (SIK-3), a key regulator of mitogenic activity (19), while nuclear factor of activated T cells 5 (NFAT5) signaling upregulates VEGF expression in breast cancer cells, thereby facilitating cancer metastasis (20). However, recent studies have indicated that HSD may have a paradoxical role in cancer treatment. It has been demonstrated to enhance the effector function of CD8+ T cells (21), reduce the expansion and accumulation of MDSCs in the blood, spleen, and tumors (22), and increase the function of NK cells by affecting the intestinal flora, specifically *Bifidobacterium (* 23). These effects promote the transformation of immunosuppression to immunogenicity. Thus, the role of HSD in cancer process appears to be double-edged.

A comprehensive review of the relationship between HSD and cancer progression, as well as its impact on tumor microenvironment reshaping, gut microbiome regulation, and the role of iron channel, is currently lacking. This paper aims to summarize the role of HSD in cancers progression. The intricate relationship between diet, the immune system, and cancer progression offers a fertile ground for further investigation, with the potential to yield innovative immunotherapeutic strategies. Gaining insight into suitable diet patterns for both health individuals and cancer patients, as well as the role of immunotherapy efficacy, is crucial in this regard.

## 2 HSD is a double sword in tumor treatment strategies

### 2.1 HSD promote cancer process

**Figure 1:**
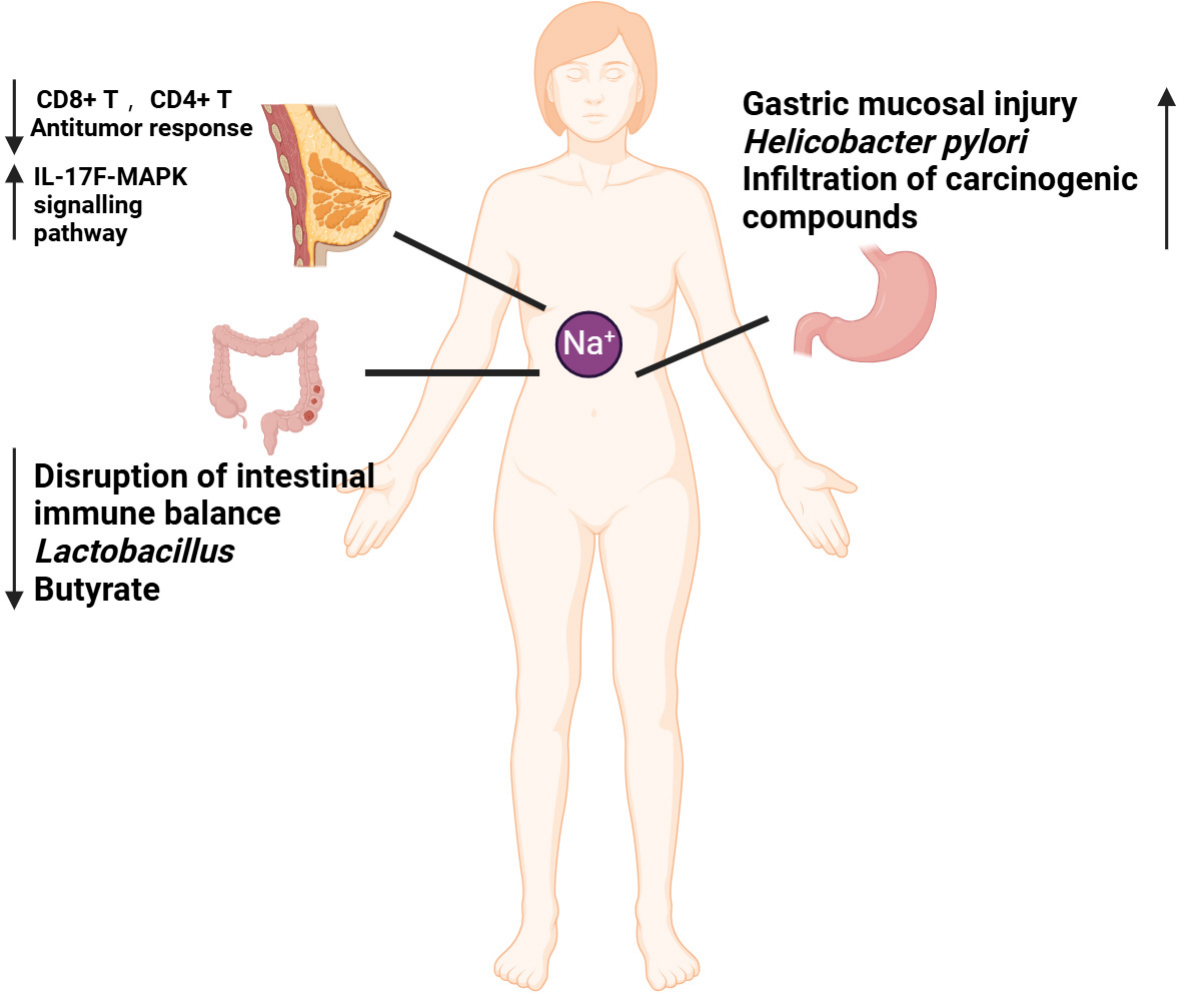
Mechanisms by which high-salt diet (HSD) promotes cancer development. The diminished capacity of CD4^+^T and CD8^+^T to eradicate tumors in the context of HSD may be attributable to an immune deficiency resulting from HSD. HSD has been demonstrated to increase the number of circulating Th17 cells. And the release of IL-17F has been observed to activate the MAPK signaling pathway, which in turn has been shown to promote the growth of breast cancer. HSD has been demonstrated to disrupt the immune balance, result in a reduction in probiotics (*lactobacillus*), and lead to a decline in favorable SCFA (butyrate), thereby promoting the development of colorectal cancer. HSD has been demonstrated to cause gastric mucosal damage, promote the increase of *H. pylori* colonization, facilitate the penetration of cancer substances, and contribute to the development of gastric cancer.

In the modern era, the relationship between dietary habits and cancer has become an increasingly prominent area of scientific research. The health effects of HSD have been extensively studied, particularly in the context of cardiovascular diseases (24) and autoimmune diseases (25). However, the relationship between HSD and cancer development is still not fully understood. After a median follow-up of 17.3 years, 485 histologically confirmed cases of epithelial renal small-cell carcinoma and 4,438 subcohort members were included in the analysis. The results indicated that higher sodium intake was significantly associated with an increased risk of renal small-cell carcinoma (26). Furthermore, prospective studies have consistently demonstrated positive associations between dietary salt intake and elevated risks of esophageal (27) and gastric cancers (28). Recent studies suggest that HSD may impact cancer progression through various mechanisms, including modulation of immune responses, changes in the composition of the gut flora, and the promotion of inflammation (**Figure 1**). It has been demonstrated that chronic HSD increase the frequency of tumor-initiating stem cells (TISCs), enhance the expression of CD80 on the surface of TGF-β-mediated TISCs, weaken the anti-tumor response of CD8+ T and CD4+ T cells, and elevate the levels of CTLA4, a marker of immune depletion, which is associated with an increase in immune depletion (29). In an *in vivo* HSD model in MMTV-PyVT mice with spontaneous tumor-forming properties, HSD was found to promote breast cancer development and lung metastasis, and to increase the frequency of circulating Th17 cells. This may contribute to breast cancer growth by activating the MAPK signaling pathway in breast cancer cells through the secretion of IL-17F (18). Research has identified that salt-inducible kinase-3 (SIK-3) is specifically upregulated in breast cancer cells exposed to high salt concentrations. SIK-3 plays a critical role in facilitating the G0/G1 phase transition of the cell cycle, thereby enhancing mitogenic activity. Additionally, it increases the surface expression of the CXCR4 chemokine receptor, which promotes tumor metastasis (19). Moreover, the production of metabolic byproducts essential for cellular building blocks is crucial. Under normoxic conditions, cancer cells predominantly undergo glycolysis, a phenomenon known as aerobic glycolysis or the Warburg effect (30). High sodium concentrations further enhance this effect in cancer cells (31). Vascular endothelial growth factor (VEGF) is a key mediator of angiogenesis (32). High salt levels increase VEGF expression in breast cancer cells via nuclear factor of activated T cells 5 (NFAT5) signaling, thereby promoting cancer metastasis (20).

Inflammatory bowel disease (IBD) has been identified as a risk factor for colorectal cancer (33). In our previous work, we identified that HSD promote DSS-induced UC process in an necroptosis-dependent manner (34). HSD have been also shown to affect the homeostasis of the intestinal mucosal barrier and to exacerbate colitis by inducing dysbiosis in the intestinal flora. This occurs through the reduction of beneficial bacteria, such as *Lactobacillus*, and the decrease in the concentration of butyrate, a short-chain fatty acid (SCFA) that is crucial for maintaining intestinal immune homeostasis. Previous studies have demonstrated that butyrate has a protective effect against colitis (35). HSD is also a significant risk factor for gastric cancer. At elevated salt concentrations, the levels of VacA toxin secreted by *Helicobacter pylori* significantly increase. VacA is a critical virulence factor that can compromise cellular membranes, resulting in cellular damage and apoptosis (36). HSD has been shown to irritate the gastric mucosa, leading to atrophic gastritis, increased DNA synthesis and cellular accretion, promotion of *H. pylori* colonization and penetration of other carcinogenic compounds, and an increased risk of gastric carcinogenesis (37, 38).

### 2.2 Potential mechanisms of HSD in antitumor response

#### 2.2.1 Tumor microenvironment

**Figure 2:**
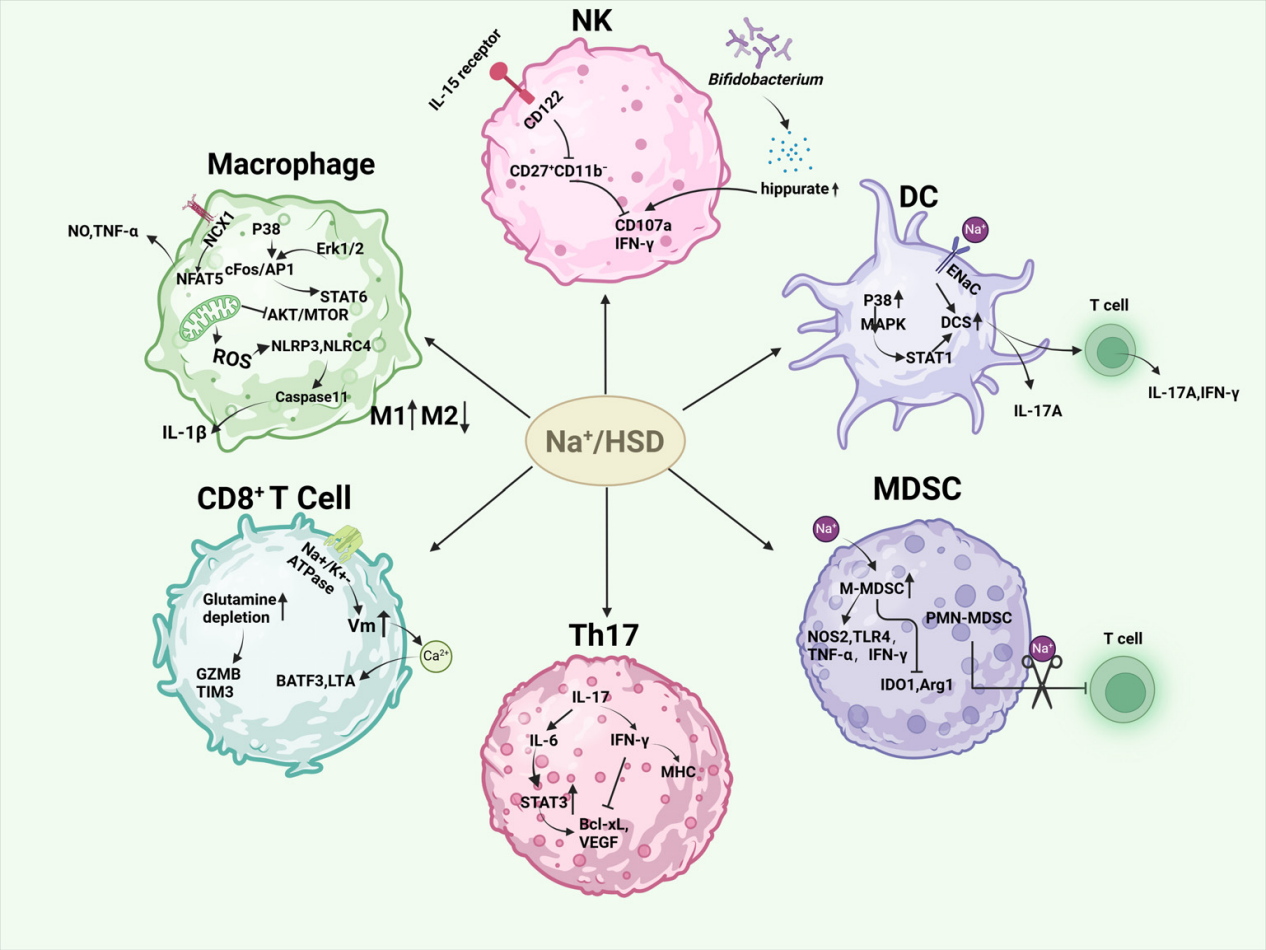
Effects of high salt or sodium ions on immune cells in the tumor microenvironment. HSD induced M1-type macrophages and inhibited M2-type macrophages by increasing the release of NO, TNF-α, and IL-1β, hindering mitochondrial metabolism in M2-type macrophages and the AKT/mTOR signaling pathway. HSD decreased the sensitivity of NK cells to IL-15 and the expression of self-cd122, hindering the proliferation, activation, and function of NK cells. In another study, HSD was able to increase *Bifidobacteria* colonization into tumors and increase their anti-tumor capacity. HSD promotes DC maturation and enhances their secretion of pro-inflammatory cytokines via P38/MAPK-STAT1, epithelial sodium channel (ENaC). HSD enhances the anti-tumor capacity of CD8^+^ cells, and the CD4^+^ subset, Th17 is increased in response to HSD. Th17 has different roles depending on the disease background. HSD facilitated the conversion of M-MDSC to pro-inflammatory and anti-tumor phenotypes, and alleviated the inhibitory effect of PMN-MDSCs on T cell function.

The term “tumor microenvironment” (TME) refers to the local environment in which a tumor grows. This environment contains a variety of elements, including cancer cells, cancer-associated fibroblast cells (CAFs), surrounding tissue cells, blood vessels, different immune cells, extracellular matrix, and various bioactive molecules. The ongoing interaction between tumor cells and the tumor microenvironment is a critical factor in tumor genesis, progression, metastasis and response to therapy (39). Cancer cells can influence their microenvironment by secreting a range of cytokines, chemokines and other factors. This leads to the reprogramming of surrounding cells, which then promote tumor growth and survival (40). HSD has been shown to promote inflammation and immune suppression within TME, which may facilitate cancer growth and metastasis. Additionally, HSD may disrupt the balance of beneficial and harmful bacteria in the gut, leading to changes in the production of metabolites that can affect the tumor microenvironment. These changes may further promote the development and progression of cancer (**Figure 2**).

A high level of macrophage infiltration is associated with a poor prognosis in many types of tumor, including breast cancer, gastric cancer, lung cancer, liver cancer and others (41–43). In some cases, macrophages can make up as much as 50% of the tumor mass (44), indicating a significant contributor of macrophages in tumor development. Macrophages can be classified into two distinct polarization states: the pro-inflammatory M1 state and the immune-suppressive M2 state (45). In the initial stage of tumor development, M1 macrophages are the predominant type. However, as the tumor progresses, there is a gradual polarization of M1 macrophages to M2 macrophages. An increase in the number of M2 macrophages is indicative of poor prognosis (45, 46). The high proportion of M2 macrophages in tumors renders macrophage-mediated killing of cancer cells ineffective in the TME, thereby facilitating tumor growth, proliferation, angiogenesis, metastasis, and epithelial-mesenchymal transition (EMT) (47). High-salt intake has been shown to have dual effects on macrophages. On one hand, the generation of mitochondrial reactive oxygen species (mtROS) under hypertonic conditions activates both NLRP3 and NLRC4 inflammasomes, resulting in elevated IL-1β production through a Caspase-1-dependent pathway (48, 49). Furthermore, after stimulation of macrophages with 500 mM NaCl (about 3% NaCI) for 8 h, secretion of IL-1β and il-18 was detected, and IL-1β release was inhibited after knocking down NLRP1 (50). This promotes the function of M1 macrophages. On the other hand, high salt intake can inhibit the function of M2 macrophages. This is achieved by impeding mitochondrial metabolism and the AKT/mTOR signaling transduction pathway in M2 macrophages (25, 51). Additionally, the NCX1 protein on macrophages responds to increased levels of extracellular sodium (Na^+^) irons concentrations. This response activates the downstream NFAT5 pathway, which lead to an increase in the expression and production of nitric oxide (NO) and tumor necrosis factor (TNF). As a result, there is an enhancement of M1-type macrophage function (52, 53). Furthermore, studies have shown that high-salt concentrations can have been demonstrated to induce M1 and proinflammatory characteristics through the p38/cFos/AP1 and Erk1/2/cFos/AP1 signaling pathways. In addition, it also inhibited the M2 type through the Erk1/2/signal transducer and activator of transcription 6 (STAT6) pathway (54).

Natural killer (NK) cells are the prototype members of innate lymphoid cells (ILC1) and can be categorized into two distinct subsets: CD56hi CD16^+^/− and CD56low CD16hi. These subsets are distinguished by their CD16 and CD56 expression levels, as previously mentioned. CD56hi CD16^+^/^−^ NK cells have been observed to secrete inflammatory cytokines. CD56lo CD16hi NK cells specialize in cytotoxic functions and cell-mediated killing. Notably, NK cells are also play a crucial role in limiting tumor metastasis and eliminating malignant cells during cancer progression (55). A study demonstrated that HSD impeded the proliferation, activation, and functionality of NK cells in mice. Specifically,HSD reduces the expression of CD122 in NK cells and inhibits the response to interleukin IL-15, thereby hindering NK cell maturation in the spleen and bone marrow (56). In contrast, another study observed that HSD induced natural killer (NK) cell-mediated tumor immunity by suppressing the expression of programmed death-1 (PD-1) while enhancing interferon gamma (IFN-γ) production and serum hippurate levels. These findings suggest that high salt has different effects on mouse NK cells depending on the disease context. Given the immunosuppressive nature of TME, numerous cytokines have the potential to impair NK effector function (23). Therefore, investigating the impact of HSD on NK cells in various disease states could provide a promising avenue for the treatment and prevention of cancer.

Dendritic cells (DCs) play a crucial role in boosting protective immunity by initiating pathogen-specific T cell responses. In order to effectively stimulate adaptive immune responses, dendritic cells must first recognize, capture, and present antigens, then up-regulate costimulatory molecules, produce inflammatory cytokines, and finally migrate to secondary lymphoid organs to present antigens to T cells (57). In the context of cancer, DCs are widely known as tumor-infiltrating dendritic cells (TIDCs). The immunogenic or tolerogenic nature of TIDC depends on the environmental signals present (58). Tumors often reprogram their surrounding microenvironment to support their own survival. The immunosuppressive factors secreted by tumors, including vascular endothelial growth factor (VEGF), IL-10, transforming growth factor β (TGF-β), prostaglandin E2 (PGE2), and other cytokines, can influence the transcriptional and metabolic activities of enzymes and proteins like: IDO, Arg1, iNOS, and STAT3 (59). This results in changes in DC metabolism, metabolites production energy transfer, and/or the accessibility of chromatin. It also inhibits DCs maturation into immunogenic cells and promotes their development into a tolerogenic phenotype (60). However, studies have shown that high salt concentrations can stimulate the maturation of mouse dendritic cells (DCs) and enhance their secretion of pro-inflammatory cytokines. It has been demonstrated that mice with systemic lupus erythematosus (SLE) exposed to high levels of salt can activate the dendritic cell system (DCS) through the p38/MAPK-STAT1 pathway (61). Similarly, excess sodium can enter DCS via sodium-sensitive channels, including the epithelial sodium channel and the α and γ subunits of the Na^+^-H^+^ exchanger. This leads to the production of immunogenic isolG-protein adducts and interleukin-1β (IL-1β), and promotes the production of cytokines IL-17A and interferon-γ (IFN-γ) by T cells (62).

T cells play a crucial role in tumor immune surveillance and immune editing. tumor cells can be identified and destroyed by T cells through the expression of specific antigens. CD8^+^ T cells, particularly cytotoxic T lymphocytes (CTLs), identify antigen peptide complexes presented by MHC-I molecules on the surface of tumor cells through their T cell receptors (TCR). After recognizing these complexes, CD8^+^ T cells eliminate tumor cells by secreting perforin and granzyme (63–65). However, tumors can impede the cytotoxic capacity of CD8^+^ cells by influencing the tumor microenvironment (TME). On the one hand, the local concentration can be regulated and a profound immunomodulatory effect on T cell behavior produced through the metabolites produced by the tumor, including spermidine, glutamate and kynurenine. This can include the induction of T cell exhaustion, the impairment of cytotoxic activity and the alteration of T cell metabolism (66). Additionally, regulatory T cells (Tregs) within the TME can inhibit the functionality of CD8^+^ T cells through the secretion of transforming growth factor-β (TGF-β) and other inhibitory cytokines (67). Interestingly, studies by Scirgolea et al. demonstrated that the addition of NaCl to CD8^+^ T cell cultures resulted in the differentiation of effector cells, the production of interferon-γ (IFN-γ), and cytotoxicity, while preserving the gene networks responsible for stem-like plasticity. HSD has been demonstrated to inhibit terminal differentiation and enhance the effector capacity of CD8^+^ T cells in a CD8^+^ T cell-dependent manner, thereby inhibiting tumor growth (21). Furthermore, elevated sodium levels alter the activity of alterations in sodium-potassium pump enzyme, leading to an increase in membrane potential (Vm) hyperpolarisation and calcium ion influx. This, in turn, promotes the up-regulation of the BATF3 gene and LTA gene, which encode for cytotoxic effector proteins. This enhances the metabolic adaptability, vitality, and memory development of CD8^+^ T cells, and improves T cell metabolic adaptability and cytotoxicity (68). Furthermore, the number of CD4^+^ T cells was increased in response to HSD, with these cells predominantly expressing IL-17, suggesting an augmentation of the CD4^+^ subtype cells Th17 (69, 70). Nevertheless, the impact of Th17 cells on tumors is intricate and multifaceted. On the one hand, IL-17 produced by Th17 has been demonstrated to promote angiogenesis, which can facilitate the progression of human hepatocellular carcinoma (HCC) (71). Additionally, it induces the production of IL-6, which then activates the oncogenic signal STAT3, leading to the up-regulation of anti-apoptotic and angiogenic genes (72). Muranski and colleagues demonstrated that IFN-γ-dependent production of tumor-specific Th17 cells inhibited tumor growth in a B16 melanoma mouse model. IFN-γ has direct pro-apoptotic and anti-angiogenic effects, activates innate immunity, and upregulates the expression of MHC molecules on tumors (73). It also increases immunogenicity and susceptibility to immune-mediated lysis. These results suggest that Th17 cells obtain the ability to exert an indirect anti-tumor effect by recruiting other tumor-specific immune cells and/or promoting anti-tumor immune responses (74, 75).

Myeloid-derived suppressor cells (MDSC), an intrinsic part of the myeloid lineage, are a heterogeneous population consisting of myeloid progenitors and precursors of myeloid cells. On the one hand, MDSC can support tumor growth by providing growth factors such as vascular endothelial growth factor (VEGF) (76), on the other hand, they can inhibit the antitumor function of various other immune cells, such as CTL, Th1, and B cells, dendritic cells (DC) and natural killer cells (NK), through various inhibitory molecular mechanisms, thereby promoting an immunosuppressive states (77, 78). MDSCs can be divided into M-MDSCs (monocytic myeloid-derived suppressor cells) and PMN-MDSCs (polymorphonuclear myeloid-derived suppressor cells). In the tumor microenvironment, M-MDSCs suppress T-cell responses by secreting immunosuppressive molecules such as IL-10 and TGF-β, as well as arginase 1 (ARG1) and nitric oxide (NO) (79). Increased ARG1 activity leads to increased L-arginine catabolism and then modulated T cell anti-tumor response (80). In addition, MDSC can consume tryptophan via IDO (81), which damages T-cell function by consuming non-essential amino acids that are crucial for T cell function. However, increased NO activity leads to an increase in ROS, which not only plays an important role in MDSC oxidative stress but also catalyzes the nitration of TCR/CD8 molecules, preventing TCR/MHC peptide interactions (82–84). However, HSD can promote the accumulation of NaCl in tumors and increase tissue osmolality. This leads to the transformation of M-MDSC into a pro-inflammatory, anti-tumor phenotype by increasing the expression of NOS2, TLR4, TNF-α and IFN-γ and decreasing IDO1 and ARG1. In addition, the number of M-MDSCs in the HSD group was significantly higher than in the control group. Although the number of PMN-MDSCs did not change, PMN-MDSCs isolated from the HSD group lost the function of inhibiting T cells, but increased the ability of T cells to proliferate (22). HSD significantly inhibits tumor growth in two independent murine tumor transplantation models, and this effect is largely independent of adaptive immune cells, but by modulating the function of MDSCs. HSD can activate the p38/MAPK-NFAT5 regulatory axis, promote the differentiation of M-MDSCs into anti-tumor macrophages and enhance anti-tumor immune responses (22, 85).

Overall, the discovery of the anti-tumor capability of a high-salt diet (HSD) has challenged the traditional view of high salt’s role in tumor progression. It highlights the dual role of HSD in biological function, as it can both promote and suppress tumor growth depending on the context. This section summarizes the novel anti-tumor response of high sodium in the tumor microenvironment (TME). This novel finding suggests that HSD may have therapeutic potential in cancer treatment, although further research is needed to fully understand its mechanisms and potential side effects.

#### 2.2.2 Gut microbiome

Gut microbiota refers to the collection of microorganisms, including bacteria, viruses, fungi, and other microbial species, that reside in the human gut (86). In recent years, a significant amount of evidences have shown that the commensal (or symbiotic) microorganisms in the human body are the crucial factors that can influence health or pathological conditions. A range of diseases, such as inflammatory bowel disease (87), coronary atherosclerosis (88), diabetes (89), non-alcoholic fatty liver disease (90), and others, have been linked to flora dysbiosis. Furthermore, gut microbiota can also interact with immune cells with metabolic to alter the immunogenicity of tumor cells, thereby compromising the anti-tumor effect of the host’s immune system (91–93). Additionally, gut microbiota can also cause an imbalance in DNA mismatch repair and increase the frequency of DNA mutations, thereby increasing the incidence of cancer (94). For example,*Shigella flexnei* induces P53 degradation in host cells through the secretases IpgD and VirA, increasing the frequency of DNA mutations (95).

The high-salt intake can result in alterations to the composition of the intestinal flora, which may subsequently lead to an imbalance in the intestinal ecosystem. It has been reported that in high-salt animal models, an increase in the relative abundance of *Alloprevotella, Prevotella 9, Allobaculum, Turicibacter*, and *Parasutterella spp* was observed. Conversely, the relative abundance of *Prevotella NK3B31, Oscillibacter, Pseudoflavonifractor, Clostridium XIVa, Johnsonella*, and *Rothia* was decreased (96–98). Several studies have demonstrated that mice with HSD exhibit a higher ratio of *Trichomonas* to *Bacteroidetes* (F/B). The F/B ratio is a marker for the evaluation of intestinal health. A ratio that is either too high or too low indicates an imbalance in the intestinal ecosystem (99, 100).

The impact of HSD on the gut microbiota and metabolites is multifaceted, with implications for cancer development. In a BALB/c mouse model, lactic acid bacteria were found to stimulate dendritic cells (DC) to secrete IL-12, which in turn, inhibits the formation of skin cancer (101). Furthermore, the derived “ferrochromium” has been shown to be directly activated through the JNK pathway, leading to tumor cell apoptosis (102). In human and mouse models of colorectal cancer, *B. fragilis* has been observed to induce apoptosis of intestinal epithelial cells in the ileal crypt and to recruit TFH, which interfered with proximal colon tumors and inhibited tumor growth in an IL-1R and IL-12-dependent manner (103), However, on a high-salt diet, the abundance of *lactobacilli* and *Bacteroides fragilis* decreased (35, 98), while *bifidobacterium* increased, leading to increased intestinal permeability and enhanced NK cell function, which promoted tumor regression (23). Furthermore, HSD can also contribute to the development of tumors by influencing the metabolites produced by the gut microbiota. Short-chain fatty acids (SCFAs) such as butyrate, propionate, and acetate are produced by the gut microbiota. It has been demonstrated that propionate and butyrate induce autophagy in human colon cancer cells and increased phosphorylation of AMPKα, leading to cellular ATP depletion and excessive production of reactive oxygen species (ROS) by reducing mTOR activity and enhancing AMP kinase activity (104). The absolute concentrations of acetate, propionate, and butyrate in the faecal samples of mice on the HSD were found to be lower than those in the control group, and the abundance of *Firmicutes* and *Bacteroidetes*, which are responsible for SCFA production, was also observed to be reduced (105, 106). Trimethylamine oxide (TMAO) is a co-metabolite of choline and carnitine produced by the gut microbiota and host diet. Elevated plasma TMAO concentrations were observed in mice exposed to 2% sodium chloride (NaCl) for a period of two weeks, and these concentrations were correlated was decreased urinary excretion of TMAO (107). TMAO produced by the gut microbiota has been observed to stimulate anti-tumor immunity mediated by CD8^+^ T cells and improve the efficacy of immunotherapy in mice with triple-negative breast cancer. Furthermore, patients with elevated TMAO levels in tumor tissue or blood exhibit enhanced responses to immunotherapy. The mechanism by which TMAO enhances CD8^+^ T cell-mediated anti-tumor immunity *in vivo* is through the induction of tumor cell pyroptosis mediated by the activation of ER stress kinase PERK (108). Analysis of 16S rDNA gene sequencing demonstrated that HSD altered the faecal microbiome, leading to a depletion of *Lactobacillus* and a significant reduction in the content of indole lactic acid (ILA) and indole acetic acid (IAA) in the faeces of mice, while the content of IAld remained unaltered (98). ILA interacts with the chromatin insulator protein CTCF of CD8^+^ T cells, resulting in the reduction of SAA3 expression, an important gene regulating cholesterol metabolism in CD8^+^ T cells. This, in turn, leads to a decrease in the cholesterol level of CD8^+^ T cells, an enhancement in the function of tumor-infiltrating CD8^+^ T cells and an inhibition of tumor growth (109).

**Figure 3:**
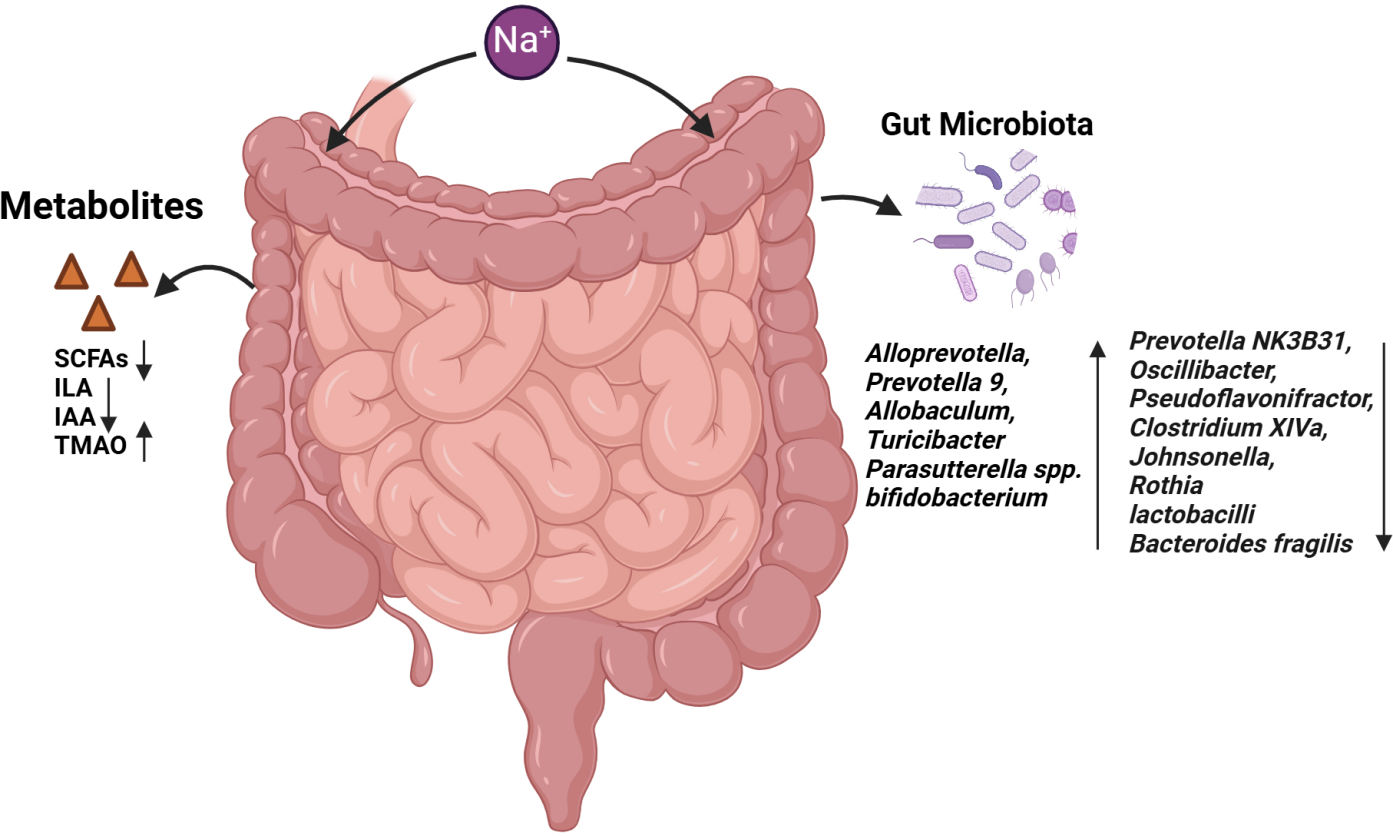
Effects of HSD on gut microbiota and gut metabolites. The administration of HSD resulted in a notable alteration in the composition of the intestinal microbiota. This led to an increase in the abundance of several bacterial species, including *Alloprevotella, Prevotella 9, Allobaculum, Turicibacter, Parasutterella* spp. and *Bifidobacterium*. Conversely, the prevalence of *Prevotella NK3B31, Oscillibacter* and *Clostridium XIVa* declined. The abundance of bacteria such as *Faecalibacterium, Pseudoflavonifractor, Clostridium XIVa, Johnsonella, Rothia, Lactobacilli* and *Bacteroides fragilis* was found to be reduced. Furthermore, the metabolites produced by the intestinal microbiota, including SCFAs, ILA and IAA, were observed to decline, while TMAO levels increased.

In conclusion, there is a complex interplay between HSD, gut microbiota and cancer (**Figure 3**). HSD may influence tumor occurrence and development by altering the composition and function of the gut microbiota. These effects may be achieved by regulating the host immune response and metabolic pathways.

#### 2.2.3 Sodium ion channel

A significant amount of attention has been devoted to the reprogramming of tumor metabolism, particularly the reprogramming of glucose metabolism, which is also known as the “Warburg effect”. This effect describes the tendency of tumor cells to produce energy through the glycolysis pathway, even in the presence of sufficient oxygen. This metabolic shift is crucial for tumor cells because it allows them to obtain energy and promote their abnormal growth, proliferation, and metastasis (110). In this process, sodium-glucose co-transporters(SGLT) play a pivotal role, utilizing the electrochemical potential energy of sodium ions to facilitate glucose transport against the concentration gradient (111). Specifically, the expression of SGLT1 in colorectal adenoma and colorectal cancer tissues has been shown to increase a progressively. Furthermore, the positive expression rate of SGLT1 protein in the adenocarcinoma group is significantly higher than that in other groups, indicating that SGLT1 may be involved in the occurrence and development of colorectal polyp cancerogenesis (112). Furthermore, according to the chemo-osmotic hypothesis, the synthesis of ATP, the main source of cellular energy, in mitochondrial is driven by an electrochemical gradient of protons across the inner mitochondrial membrane (113). Recent studies have shown that this process also involves the transport of sodium ions, which are exchanged for protons by mitochondrial complex I. This results in a sodium gradient parallel to the proton gradient, accounting for half of the mitochondrial membrane potential and being essential for ATP production (114). The processes of sodium ion transport and maintenance are inextricably related to the sodium ion channels that facilitate these functions. Numerous studies have indicated that sodium channels are highly expressed in cancerous cells, thereby suggesting a potential correlation between sodium channels and cancer development (115–117).

The Na^+^/K^+^ ATPase is a crucial transmembrane protein responsible for maintaining the concentration gradient of sodium and potassium between the intracellular and extracellular environments. Studies have shown that the expression level of the alpha 1 subunit of the sodium-potassium pump (*ATP1A1*) is linked to tumor progression and clinical outcome in gastric cancer (GC). Specifically, high expression level of ATP1A1 is associated with poor prognosis in gastric cancer patients, and knockdown experiments on GC cell lines have shown that inhibiting ATP1A1 can suppress cell proliferation and induce apoptosis (118). Furthermore, the sodium-potassium pump inhibitor ouaben has been found to inhibit the growth and migration of glioma U-87MG cells by inhibiting the Akt/mTOR signaling pathway and downregulating HIF-1α expression (119).

Sodium-dependent glucose transporters (SGLTs) are generally responsible for active glucose uptake in the kidney and are also functionally expressed in many types of cancers (120, 121). In addition, the electrically neutral Na^+^-K^+^-Cl- cotransporter (NKCC), which is a crucial regulator of osmotic balance and cell volume that facilitates Na^+^, K^+^ and 2Cl^-^ transport into cells. This cotransporter is also upregulated in many cancers (122). This combination of these nutrient and electrolyte transport mechanisms not only depends on Na^+^ homeostasis in the tumor microenvironment, but also enhances Na^+^ influx. This, in turn, leads to the increased Na^+^ signaling tumors. Furthermore, Na^+^ channels expressed on tumor cells also allow Na^+^ influx and Na^+^ efflux.

Voltage-gated Na^+^ channel (VGSC) proteins are commonly expressed in electrically excited cells, where they trigger action potentials via Na^+^ influx. These proteins are expressed in many tumor cell types, and promote cancer cell invasion and metastasis (123, 124). Although the voltage-dependent opening of these channels is transient, they also conduct a “sustained” inward Na^+^ current under resting conditions, a mechanism that provides a pathway for Na^+^ to enter the cytoplasm of non-excited tumor cells (124, 125).

Amiloride-sensitive epithelial Na^+^ channels (ENaC) and related acid-sensitive ion channels (ASics) are both Na^+^ selective ion channels, which allow voltage independent inward Na^+^ currents. Both ENaC and ASIC have been implicated in the proliferation, migration, invasion, and metastasis of various cancers (126, 127). Na^+^ flux through ENaC and ASIC is regulated by extracellular H^+^ (128). Therefore, in an acidic environment, both channels may contribute to increased intracellular Na^+^ concentrations in tumor cells. In addition, the N-methyl-D-aspartate receptor (NMDAR) can also increase the concentration of Na^+^ influx into tumor cells. These ligand-gated, non-selective cation channels are normally expressed in the central nervous system (CNS) and are activated by the neurotransmitter glutamate. NMDARs are expressed in many tumor types, including non-neuronal tumors such as pancreatic, breast and ovarian cancers, where they regulate invasion and are associated with poor prognosis (129–131).

Proteins that form G protein-coupled receptor-activated Na leak channels (NALCN) have been proposed as potential sites of cancer susceptibility (132). Indeed, NALCN-mediated sodium influx into cancer cells maintains intracellular calcium oscillations through specific ion transporter chains. And then, a series of signaling cascades that promote Src kinase activity, actin remodeling, and proteolytic enzyme secretion of the NACLN-colocalized proto-oncogene. These processes increase thereby increasing cancer cell invasion potential and metastatic lesions *in vivo* (133). Additionally, the two-pore (TPC) family of lysosomal and endosomal cation channels can increase cytosolic Ca^2+^ and Na^+^ concentrations and have been shown to promote lung cancer cell migration (134), epithelial-mesenchymal transition in breast cancer cells (135) and hepatocellular carcinoma cell proliferation (136).

**Figure 4:**
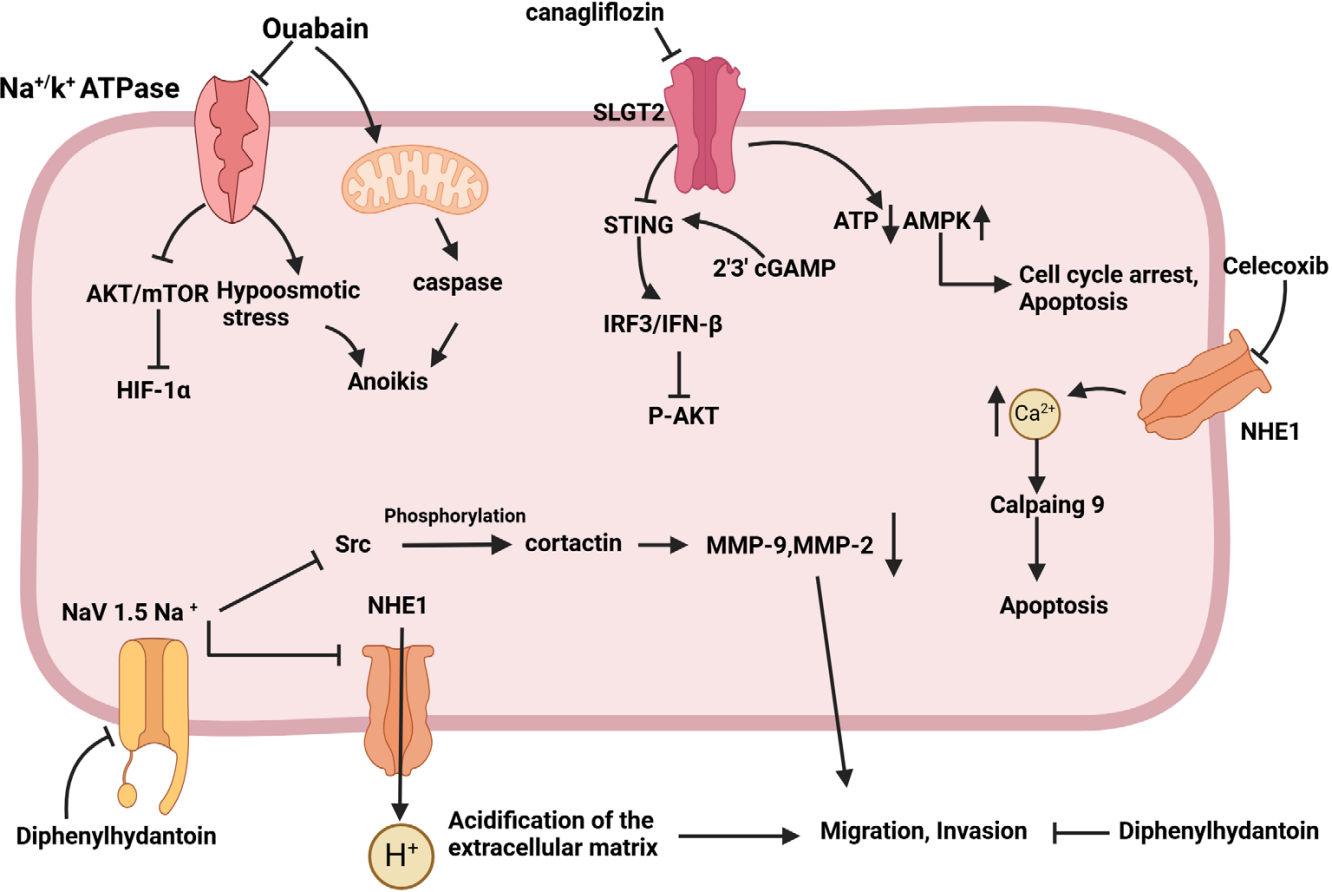
Schematic of the mechanism by which sodium channel inhibition exerts anti-tumor effects. Ouabain activates caspase through the mitochondrial pathway, induces hypoosmotic stress by inhibiting the Na^+^/K^+^ ATPase pump, sensitizes cells to anoikis, and inhibits tumor cell metastasis. Canagliflozin has the capacity to down-regulate oxidative phosphorylation, reduce the intracellular concentration of ATP, up-regulate AMPK phosphorylation, and induce cell cycle arrest in the G1/G0 phase, as well as apoptosis. The objective is to stimulate STING expression, activate the IRF3/IFN-β pathway, and ultimately inhibit AKT phosphorylation and tumor growth. Celecoxib was observed to downregulate NHE-1, increase Ca^2+^ content, and induce apoptosis via activated calpain9. Diphenylhydantoin has been demonstrated to target the Nav1.5 channel, reducing the content of MMP9 and MMP2, inhibiting the acidification of the extracellular matrix, and reducing the migration and invasion ability of tumors.

Sodium channels play multiple roles in tumor cells, not only supporting basic cellular functions, but also being closely associated with tumor invasiveness and metastasis. Currently, various pharmacological agents have demonstrated anti-tumor activity by inhibiting sodium channels (**Figure 4**) (121, 137–139). These findings hint that sodium channel blockers may represent a novel class of anti-tumor agents, capable of inhibiting tumor cell proliferation and invasion by modulating sodium channel activity. This presents a promising direction for the development of new therapeutic strategies and potential drug targets for cancer treatment.

In total, The role of HSD in tumor development is complex and showed a double-sword role in tumorigenesis, as outlined in **Table 1**. While HSD can have certain inhibitory effects on tumor growth by stimulating the release of inflammatory factors from immune cells, which can transform the immunosuppressive tumor microenvironment into an immunogenic TME, they also have other effects. Specifically, HSD can modulate the composition of the gut microbiota and enhance intestinal permeability. This allows the migration of specific gut microbes, such as Bifidobacteria, to localize within the tumor, which in turn enhances immune cell function. Additionally, HSD can also impact the oxidative stress, epigenetic change, and hormonal imbalances, and other things.

**Table 1:** Complex role of high salt diet in tumorigenesis.

Aspect	Type	Mechanism	Functions	Reference
Promotion of Gastric Cancer	Gastric Cancer	*H. pylori* colonization**↑**	Pro-tumor	(28)
Inflammatory Responses	Melanoma	CD8^+^ T cell effector functions**↑** MDSCs function, growth**↓**	Anti-tumor	(68)
				(68)
Oxidative Stress	Pancreatic Cancer	Inflammation and oxidative stress↑	Pro-tumor	(140)
Alteration of Gut Microbiota	Colorectal Cancer Melanoma	*Lactobacillus*,butyrate IBD*↑* *Bifidobacterium*,gut permeability, NK cells ability↑	Pro-tumor Anti-tumor	(35) (23)
Hormonal Imbalances	Colorectal Cancer	IL-17A and iNOS,colonic polyps↓	Anti-tumor	(141)
Renal Function Impairment	Renal Cell Carcinoma	Hypertension↑	Pro-tumor	(26)
Epigenetic Changes	Breast Cancer	T cell differentiation, cytotoxicity, IFN-γ production↑ MDSCs differentiation T cell anti-tumor response.↑	Anti-tumor	(21)
				(22)
Interaction with Other Factors	Breast Cancer	Tumor-initiating stem cells (TISC),TGF-βR2 and CD80↑	Pro-tumor	(29)

## 3 Discussion

This article provides a comprehensive review of how a high salt diet (HSD) influences cancer development and the immune microenvironment through various biological pathways. Chronic high salt intake in daily life continuously stimulates the body’s immune system, leading to an imbalance that skews towards anti-inflammatory and pro-tumor responses. As clinical correlation studies have indicated, HSD is one of the risk factors associated with tumor development. However, in the context of specific diseases, short-term high salt intake can transiently enhance immune function, such as by inhibiting the differentiation of myeloid-derived suppressor cells (MDSCs), promoting the differentiation and anti-tumor activity of CD8^+^ T cells, promoting the proliferation and activation of NK cells and improving their killing ability to tumor cells. Moreover, inflammatory factors induced by high salt intake can shift the tumor microenvironment towards a pro-inflammatory state, which may inhibit tumor progression. Therefore, HSD can be developed as one of the treatments for cancer patients in the future, but salt intake and when to adopt HSD treatment strategies need to be carefully considered in the development of treatment strategies.

The relationship between the gut microbiome and human cancer constitutes a complex and multifaceted field wherein these microorganisms influence tumor development through host interactions, modulate immune system maturation, and impact systemic responses. Alterations in the intestinal flora are associated with tumor development. Probiotics can exert anti-tumor effects by producing short-chain fatty acids (SCFAs) and activating the immune system. Moreover, prebiotics and microbial metabolites may enhance tumor therapeutic efficacy by modulating the composition and function of gut microbiota. Probiotics play a crucial role in maintaining the homeostasis of the intestinal flora. On the one hand, probiotics can inhibit the growth of harmful bacteria by competing for nutrients and adhesion sites, thereby promoting the proliferation of beneficial bacteria (such as *Lactobacillus*, *Bifidobacterium*, etc.) in the gut and maintaining the balance of intestinal flora. On the other hand, probiotics can increase the proportion of beneficial bacteria and reduce the number of opportunistic and pathogenic bacteria to improve the structure of intestinal flora. Observations have shown that HSD can decrease the abundance of certain probiotics like *Lactobacillus* while increasing levels of *Bifidobacteria* under specific conditions, which exhibit anti-tumor properties. These findings suggest that a “high-salt diet” may have context-dependent effects on different disease states. Probiotic therapy has demonstrated potential to reverse some adverse effects of HSD on the gut microbiome, including reductions in Th17 cell counts. However, there is insufficient evidence to support the notion that probiotic therapy can mitigate the effects of HSD on tumor progression. Future studies should further elucidate these interactions and verify whether probiotic therapy can be used as a new therapeutic strategy for cancer prevention and treatment.

Ion channels are crucial in the initiation, progression, and metastasis of tumors. They modulate key biological processes such as cell proliferation, apoptosis, invasion, and metastasis by regulating ion flux across cellular membranes. Elevated sodium concentrations in tumor tissues and aberrant expression of sodium channels in various cancer cells indicate a significant correlation between these factors and tumor development. Currently, inhibitors targeting sodium-related channels have demonstrated significant inhibitory effects on tumor progression. Developing specific targeted therapies for distinct subtypes of sodium channels may represent a promising therapeutic strategy. Sodium channels play a crucial role in tumor progression through the modulation of sodium ion flow and the activation or inhibition of various downstream signaling pathways. Therefore, an in-depth investigation into the mechanisms of sodium channels and their associated signaling pathways in tumors holds significant potential for developing novel anti-tumor therapeutic strategies.

In conclusion, future research is needed to elucidate the specific mechanisms by which HSD impacts tumor initiation, progression, and immune regulation. It is also important to fully consider the influence of individual differences and pathological conditions when studying these effects. Building on this understanding On this basis, the development of targeted dietary intervention strategies and new drugs could lead to more precise and effective cancer treatment plans. Additionally with the development of microbiome research, the use of microbial regulatory interventions to rationally regulate human salt intake may be an important future development direction in the field of public health.
